# A review on the role of gut microbiota in immune checkpoint blockade therapy for cancer

**DOI:** 10.1007/s00335-021-09867-3

**Published:** 2021-03-30

**Authors:** Esther Kim, Hyeok Ahn, Hansoo Park

**Affiliations:** 1 California Institute of Technology, 1200 E California Blvd, Pasadena, CA 91125 USA; 2 School of Life Science, Gwangju Institute of Science and Technology (GIST), Cheomdangwagi-ro 123, Buk-gu, Gwangju, 61005 Korea; 3 Department of Biomedical Science and Engineering, Gwangju Institute of Science and Technology (GIST), Cheomdangwagi-ro 123, Buk-gu, Gwangju, 61005 Korea

## Abstract

Gut microbiota has been studied in relation to human health and disease prediction for decades. Also, immune checkpoints (ICPs) are enthusiastically investigated for anti-tumor immunotherapy. Recent studies show potential of gut microbiome and gut cytokines as biomarkers for carcinogenesis and response prediction of immune checkpoint inhibitor (ICI) response. Evidence has revealed that intestinal microorganisms play a major role in the effectiveness of programmed cell death 1 (PD-1) and cytotoxic T lymphocyte-associated antigen 4 (CTLA-4) blockade. In this review, we have focused on how microbiome and microbiome-generated cytokines affect immune checkpoints. We have also described the molecular mechanisms behind this interplay and the bacterial strains that have a potential role in immunotherapy.

## Introduction

Immune checkpoints (ICPs) provide signals that regulate antigen recognition by the T cells, thereby preventing the immune system from attacking cells uncontrollably (He and Xu 2020). Located at the cell surface, ICPs alter the cell function upon binding the ligands secreted by other cells. Programmed cell death protein 1 (PD-1) and cytotoxic T lymphocyte-associated antigen 4 (CTLA-4) are co-inhibitory ICPs; they are expressed on activated tumor-specific CD4^+^ cells and CD8^+^ T-lymphocytes, and inhibit the progression of the immune reaction. In the absence of ICP proteins and their ligands, T cells function regularly to kill tumor cells. However, when tumor cells express the ligands for ICP proteins, they bind to the inhibitory proteins expressed on the T cells and inhibit the T cells from killing the tumor cells (Wei et al. 2018). Accordingly, the presence of an antagonist of the inhibitory signal known as the immune checkpoint inhibitor (ICI) (a corresponding antibody that inhibits the receptor function) enhances the antitumor immunity. The most commonly used inhibitors for CTLA-4 include ipilimumab and tremelimumab, while those for PD-1 include pembrolizumab and nivolumab; the inhibitors for programmed death-ligand 1 (PD-L1), the primary ligand of PD-1, include atezolimumab, avelumab, and durvalumab. The ICPs are, therefore, exploited by the tumor cells as an immune escape mechanism, suppressing the adaptive immune responses. Stimulated expression of the antigens expressed in the tumor microenvironment result in an increased expression of ICP proteins, which in turn decreases the effectiveness of the antitumor immune responses by debilitating the cytotoxic and cytokine-producing ability of the T cells (Darvin et al. 2018). Thus, PD-1 and CTLA-4 are attractive targets for anti-cancer immunotherapy.

While various biomarkers are known to reflect the tumor immune microenvironment, such as tumor mutational burden, mismatch-repair deficiency, and density of tumor-infiltrating lymphocyte (TIL), recently, both cytokines and gut microbiome have been extensively studied owing to their potential to serve as biomarkers for both cancer progression and response prediction to ICIs (Yi et al. 2018). Cytokines, released in response to infection and inflammation, play a critical role in modulating the development and progression of malignant cells by their complex and vast network. Interferon (IFN), the first discovered member of the cytokine family, which presently forms an essential part of the innate immune system, evokes anti-viral defense response against viral infections. Interleukins (ILs), another important family of cytokines, are responsible for various functions of the immune cells, including activation, proliferation, motility, and differentiation. Hence, they exhibit both pro- and anti-inflammatory properties (Zhang and An 2007). While the immunological functions of most ILs are known, many are yet to be unveiled. A few ILs have biphasic effects and some even have differences in their function depending upon the type of the tumor cell present. In practice, cytokines have been implemented as biomarkers to observe cancer cell progression in various cancer patients.

Gut microbiota has a vital role in modulating the immune system responses. Furthermore, it has been reported that the gut microbiome essentially governs the efficacy of PD-L1. In this review article, various studies revealing the effect of the microbiome on the efficacy of PD-1 and CTLA-4 blockade have been reviewed. Negative impact of dysbiosis due to antibiotics on the efficacy of PD-1 and CTLA-4 blockade was also affirmed. Fecal microbiome transplant (FMT) from patients responsive to the ICI treatment resulted in improved efficacy of ICP blockade. These studies identified several bacterial genera imparting benefits to ICI treatment. Immune mechanisms that altered the efficacy of the ICP blockade treatment involved cytokines. The related studies have been summarized in Table 1.

**Table 1:** Studies on the effect of gut microbiome on the efficacy of immune checkpoints (ICPs) through cytokines (Routy et al. 2018; Gopalakrishnan et al. 2018; Matson et al. 2018; Daillère et al. 2016; Tanoue et al. 2019; Sivan et al. 2015; Xu et al. 2020; Vétizou et al. 2015; Garris et al. 2018; Pushalkar et al. 2018)

Reference	Treatment	Bacterial strain	Cytokine	Mechanism (Effect on the efficacy of treatment)
Routy et al. (2018)	PD-1 blockade	*Akkermansia muciniphila* alone or combined with *Enterococcus hirae*	IL-12 CXCR3^+^CD4^+^ T cells IFN-ɣ CCR9	Accumulation of CXCR3^+^CD4^+^ T cells Both strains induce secretion of IL-12 by dendritic cells Cell reactivity against *A. muciniphila*, IFN-ɣ released above the median value associated with prolonged progression-free survival R CCR9 and CXCR3 accumulated 48 h after FMT in mesenteric lymph nodes
Gopalakrishnan et al. (2018)	PD-1 blockade	*Ruminococcaceae family* *Faecalibacterium* sp.	CD4^+^FoxP3^+^ T cells IL-17	Regulatory CD4^+^Foxp3^+^T cells and CD4^+^IL-17^+^ T cells in the spleen were frequently found after NR-FMT in mice, alluding impaired host immune system In the R group, Foxp3 and IL-17 levels were lower, suggesting a possible mechanism for the increased efficacy
Matson et al. (2018)	PD-1 blockade	*Bifidobacterium longum* *Collinsella aerofaciens* *Enterococcus faecium*	SIY-specific CD8^+^ T cells IFN-ɣ Batf3-lineage DCs	SIY-specific CD8^+^ T cells became abundant, but not for FoxP3^+^CD4^+^ regulatory T cells, in mice that were treated with R group-fecal samples Higher frequency of Bastf3-lineage dendritic cells and Th1 response was detected in the bacterial strains that were abundant in the R group R group had higher IFN and CD8 levels, but exhibited no change in CD4 levels
Daillère et al. (2016)	Cyclophosphamide	*Enterococcus hirae* *Barnesiella intestinihominis*	Foxp3 and/or gdT17 cells CCR6^+^ CXCR3^+^CD4^+^ T cells IFN-ɣ^+^IL-17^+^ cells NOD2	Both strains induced reduction of Treg cells in the TME (Foxp3 and gdT17 cells) The two bacteria increased IFN-ɣ levels *E. hirae* increased the frequency of intratumoral CD8 over Treg *B. intestinihominis* boosted the intrusion of IFN- γ -producing γdTIL in cancer lesions NOD2 receptor decreased the activity of both bacterial strains *E. hirae* selectively mounted pTh17 immune responses by IFN- γ^+^IL-17^+^ cells and CCR6^+^CXCR3^+^CD4^+^ T cells accumulation
Tanoue et al. (2019)	CTLA-4 blockade PD-1 blockade	*Parabacteroides distasonis* *Parabacteroides gordonii* *Alistipes senegalensis* *Parabacteroides johnsonii* *Paraprevotella xylaniphila* *Bacteroides dorei* *Bacteroides uniformis* JCM 5828 *Eubacterium limosum* *Ruminococcaceae bacterium* cv2 *Phascolarctobacterium faecium* *Fusobacterium ulcerans*	IFN-γ-producing CD8^+^ T cells CD103^+^	CD8 T cells produced IFN-γ in the intestine Bacterial mix treatment reduced induction of colonic IFN-γ^+^ CD8 T cells in *Batf3*^−/−^ mice and Xcr1-Cre × ROSA-DTA mice
Sivan et al. (2015)	PD-1 blockade	*Bifidobacterium*	CD8^+^ T cells	Heightened priming and accumulation of CD8^+^ T cells in the TME resulted in therapeutic efficacy
Xu et al. (2020)	PD-1 blockade	*Prevotella* sp. CAG:485 *Akkermansia*	IFN-γ IL-2	Alternations in the gut microbiome led to changes in GPL metabolism level, which could have influenced IFN-γ and IL-2 expression, resulting in efficacy of anti-PD-1 therapy
Vétizou et al. (2015)	CTLA-4 blockade	*Bacteroides fragilis* *Bacteroides Thetaiotaomicron Burkholderiales*	IL-12	IL-12-dependent Th1 response was beneficial for tumor control
Garris et al. (2018)	PD-1 blockade	N/A	IFN-ɣ IL-12 NF-kB	Effective anti-tumor responses required a subset of tumor-infiltrating DCs, which produced IL-12 upon sensing IFN- γ, resulting in IL-12 stimulated antitumor T cell immunity Activation of non-canonical NF-kB transcription factor enhanced IL-12 producing dendritic cells leading to sensitizing of tumors to anti-PD-1 treatment
Pushalkar et al. (2018)	–	*Proteobacteria* *Bacteroidetes* *Firmicutes* *Bifidobacterium pseudolongum*	IL-10 CD4^+^ T cells CD8^+^ T cells	Manipulation of the gut microbiome increased Th1 polarization of CD4^+^ T cells and enhanced the cytotoxic activity of CD8^+^ T cells FOXP3^+^ Treg cells facilitated the tumor immune escape in PDA Cell-free extracts from *B. pseudolongum* polarized macrophages, which upregulated IL-10

*PD-1* programmed cell death protein 1, *CTLA-4* cytotoxic T lymphocyte-associated antigen 4, *IL* interleukin, *IFN* interferon, *R* responder, *NR* non-responder, *FMT* fecal microbiota transplant, *NOD* nucleotide oligomerization domain, *DC* dendritic cell, *Th1* T helper 1, *Treg* T regulatory, *TILs* tumor-infiltrating lymphocytes, *NF-kB* nuclear factor kappa-light-chain-enhancer of activated B cells, *PDA* pancreatic ductal adenocarcinoma

## Main text

Although immunotherapy is a long-used practice, it has recently achieved exceptional success against distinct hematological and solid metastatic malignancies (Waldmann 2003). It has been reported that the ICIs disrupt the interaction between the ligands and the T cell inhibitory receptors, inducing T lymphocyte-mediated immune responses (Pardoll 2015). PD-1 blockade with its corresponding monoclonal antibodies (mAbs) is remarkably effective against advanced melanoma, non-small cell lung cancer (NSCLC), and renal cell carcinoma (RCC) (Routy et al. 2018).

### PD-1 antibody immunotherapy and the effect of FMT on PD-1 antibody efficacy

The study by Routy et al. ushered in a new perspective by connecting the field of microbiome and immunotherapy targeting PD-L1 (Routy et al. 2018). The prospect that primary resistance to PD-1 blockade may be affected by the microbial imbalance associated with antibiotic or malignant disease in tumor-bearing mice and cancer patients was investigated. The authors concluded that the aberrant composition of the gut microbiome can result in primary resistance to ICIs. The impact of antibiotics (ATBs) on cancer patients, specifically those having advanced NSCLC, RCC, or urothelial carcinoma, after they received PD-1/PD-L1 mAb treatments was studied. In the absence of ATBs, the tumor growth was significantly reduced when injected with PD-1 mAbs. However, mAbs were essentially ineffective when the mice (with RET melanoma and MCA-205 sarcoma) were previously treated with ATB. Hence, antibiotic consumption was correlated to negative response to PD-1 blockade. Based on the response evaluation criteria in solid tumors (RECIST) version 1.1 (Eisenhauer et al. 2009), patients who received PD-1/PD-L1 treatment were segregated into two groups: responders (R) and non-responders (NR). FMT from the stool samples of these patients to ATB-treated mice revealed that the stool samples from the R patients induced sensitivity, while those from NR patients induced resistance to PD-1 blockade, which is commensurate with efficiency in ATB-treated or germ-free mice. Two weeks after the inoculation with fecal samples and MCA-205 tumor cells, the ATB-treated mice were subjected to treatment with PD-1 mAbs. The mice that received FMT from R patients showed slower tumor growth than those that received FMT from NR patients. Between the two groups, the R group was enriched with several key bacterial genera and strains compared to the other. *Akkermansia muciniphila*, the most prominent commensal bacterium associated with positive clinical outcome, was found in both NSCLC and RCC patients. *Enterococcus hirae* 13144 (*E. hirae*) was also observed repeatedly in R-NSCLC patients. A combination of *A. muciniphila* and *E. hirae* led to the accumulation of central memory CD4^+^ T cells expressing chemokine receptors CCR9 (associated with small intestine) and CXCR3 (associated with T helper 1 (Th1) immune response). Increased recruitment of these CD4^+^ T cells is dependent on IL-12 secretion induced by *A. muciniphila* and *E. hirae*.

### CTLA-4 blockade immunotherapy

A similar study by Vetizou et al. revealed an IL-12-dependent Th1 immune response induced by *Bacteroides fragilis*, which resulted in an increased response to CTLA-4 blockade (Vétizou et al. 2015). The authors found that the CTLA-4 blockade-induced immune-stimulatory effects were dependent on the microbiome, which in turn was regulated by the mobilization of intestinal lamina propria CD11b^+^ dendritic cells (DC) and IL-12-associated Th1 immune response. Germ-free mice and mice treated with a combination of antibiotics, including ampicillin, colistin, and streptomycin (ACS), were re-colonized with bacterial species associated with CTLA-4 antibody-treated intestinal mucosa. In both groups of mice, reduced activation of the CD4^+^ T cells and TILs in Ret melanoma and the MC38 colon cancer models indicated significantly reduced efficacy of CTLA-4 antibody. The CD4^+^ T cells that were harvested from the spleens of CTLA-4 antibody-treated mice, or from the blood of the patients with metastatic melanoma (MM) or NSCLC after treatment with ipilimumab, demonstrated recovery of the Th1 phenotype. *B. fragilis* was observed to partially restore the efficacy of CTLA-4 antibody that was reduced due to the lack of gut microbiota. Abundance of *B. fragilis* was associated with reduced size of the tumor in the CTLA-4 blockade-treated mice. Hence, the microbiota composition influenced the efficacy of CTLA-4 blockade (*B. fragilis* and/or *B. thetaiotaomicron* and *Burkholderiales*).

### The effect of specific bacterial strains, cytokines and immune cells on immunomodulation

Garris et al. in their study reinforced the significant role of IL-12 in antitumor responses (Garris et al. 2018). The study findings revealed that the activation of antitumor T cells by anti-PD-1 is indirect, as it requires a crosstalk between the T cells and DCs. The crosstalk is, however, dependent on IFN gamma (IFN-ɣ) and IL-12. Anti-PD-1 mAbs antagonize the PD-1 inhibitory pathway in T cells and, thereby, heighten the activation of CD8^+^ T cells. In turn, the DCs sense the IFN-ɣ released from the mAb-activated T cells, instead of binding directly to the drug. DCs produce IL-12, which then stimulates the antitumor T cell immunity and sensitizes the tumors to anti-PD-1 treatment. Moreover, activation of the non-canonical nuclear factor kappa-light-chain-enhancer of activated B cells (NF-kB) transcription factor pathway augments the DCs that produce IL-12. Thus, NF-kB was identified as a potential therapeutic target for enhancing the checkpoint blockade response.

Xu et al. established the gut microbial genome and various plasma metabolites associated with PD-1 antibody immunotherapy in CT26 tumor-bearing mice. The 16S ribosomal RNA (rRNA) gene sequencing, metagenomic shotgun sequencing, and non-targeted metabolomic analyses revealed that antibiotic treatment altered the taxonomic composition of the gut (Xu et al. 2020). An abnormal gut microbiome lowered the efficacy of PD-1 antibody immunotherapy. The relative therapeutic efficacy of PD-1 antibody treatment in combination with different antibiotics varied among the experimental groups: control group (good-response), vancomycin-treated (Vanc) group (medium-response), colistin-treated (Coli) group (poor-response), and ACS-treated group mice (no-response). *Prevotella* sp*.* CAG: 485 and *A. muciniphila* were related to positive therapeutic immunotherapy efficacy. On the contrary, *Bacteroides* sp. and *Bacteroides* sp. CAG: 927 were associated with negative therapeutic efficacy. Similar to the observations by Routy et al. (2018), this study also revealed enhanced immunotherapeutic efficiency on enriching the intestine with *Akkermansia* sp. The better efficacy of PD-1 antibody was linked to the glycerophospholipid metabolic pathway, as changes in the gut microbiome altered the level of glycerophospholipid metabolism. Xu and his colleagues detected that while there was no change in the number of CD4^+^ and CD8^+^ T cells, the cell IFN-ɣ and IL-2 concentrations in the antibiotic-treated groups were significantly reduced compared to that in the control group. Thus, it was suggested that metabolism may affect the expression of immune-related cytokines, IFN-ɣ and IL-2, in the tumor microenvironment, which then alters the therapeutic effect of PD-1 antibody. In addition, the metagenomic pathway function prediction demonstrated IL-17 secretion to be linked with negative immunotherapeutic efficacy.

Sivan et al. compared the effects of anti-PD-1/PD-L1 mAbs by observing the subcutaneous B16.SIY melanoma growth in genetically similar C57BL/6 mice obtained from Jackson Laboratory (JAX) and Taconic Farms (TAC) (Sivan et al. 2015), which harbored a disparate commensal microbiota. Aggressive tumor growth observed in TAC mice resulted from an immune-mediated response. Both the accumulation of intratumoral CD8^+^ T cells and the responses of tumor-specific T cells were lower in TAC mice than that in JAX mice. Transfer of JAX fecal material into TAC mice alone was sufficient to reduce the tumor growth rate and increase the infiltration of the CD8^+^ T cells. When the JAX fecal material was combined with PD-L1, tumor control and tumor antigen-specific T cell responses were enhanced. This indicates that the composition of the commensal microbiome affects the antitumor immunity and the efficacy of anti-PD-L1 mAb. The only genus-level taxon that significantly correlated with the accumulation of activated antigen-specific T cells was *Bifidobacterium*. The strongest association was reported with antitumor T cell response, and it significantly increased with the transplant of JAX fecal material into TAC. The positive effect in JAX mice could be attributed to *Bifidobacterium*. When the mice were treated with *Bifidobacterium*, tumor control was notably enhanced. The mechanism of this therapeutic effect is rather indirect, mediated by the host antitumor T cell responses, as *Bifidobacterium* treatment led to extensive induction of tumor-specific T cells. However, *Bifidobacterium* had no effect in CD8-depleted mice. TAC mice inoculated with B16 parental tumor cells or MB49 bladder cancer cells, when subjected to treatment with *Bifidobacterium* displayed delayed tumor growth. Hence, administration of a cocktail of *Bifidobacterium* sp. in TAC mice containing melanoma was identified as a positive promoter of antitumor response. Further, in combination with ICI, tumor growth significantly decreased. In both JAX and *Bifidobacterium*-treated TAC mice, an augmented proportion of major histocompatibility complex (MHC) Class II^hi^ DCs and IFN-ɣ were reported in the tumors.

Matson et al. examined baseline stool samples from metastatic melanoma patients to evaluate the association between commensal bacterial composition and clinical efficiency of PD-1 blockade immunotherapy (Matson et al. 2018). Similar to the study by Routy et al. (2018), the patients were categorized into R and NR groups based on the RECIST 1.1 version. Three weeks after inoculation with B16.SIY melanoma cells, the R microbiota-reconstituted mice exhibited a significant increase in SIY-specific CD8^+^ T cells (thereby augmenting priming of tumor antigen-specific CD8^+^ T cells), but no change was observed in FoxP3^+^CD4^+^ regulatory T cells. IFN-ɣ enzyme-linked immunosorbent spot of ex vivo SIY-stimulated splenocytes also exhibited an increased frequency of activated T cells. While anti-PD-L1 efficacy was enhanced in mice colonized with R microbiota, mice colonized with NR microbiota exhibited completely ineffective anti-PD-L1. Slower baseline tumor growth in two of the three R-derived mouse cohorts was congruent with the findings of the study by Sivan et al. (2015) that reported FMT from R alone to be insufficient in inducing slower tumor growth. An integration of three methods (16S rRNA gene sequencing, metagenomic shotgun sequencing, and quantitative polymerase chain reaction (qPCR) for selected bacteria) revealed ten species that were differentially enriched in R versus NR groups. Eight bacterial species abundant in the R group, included *Enterococcus faecium*, *Collinsella aerofaciens*, *Bifidobacterium adolescentis*, *Klebsiella pneumoniae*, *Veillonella parvula*, *Parabacteroides merdae*, *Lactobacillus* sp., and *Bifidobacterium longum*. *Ruminococcus obeum* and *Roseburia intestinalis* were abundant in the NR group*.* This again complies with the study by Sivan and colleagues, who reported *Bifidobacteriaceae* family members to be associated with better immune-mediated tumor control and positive efficacy of the anti-PD-L1 therapy in mice. Monocolonization with the R group-enriched species resulted in decreased frequency of the colonic regulatory T cells, increased frequency of Batf3-lineage DCs, and enhanced Th1 response. Thus, the data suggests that the composition of the commensal microbiota plays a significant role in determining the therapeutic efficacy of anti-PD-1 monoclonal antibody and, hence, can be used as a biomarker for clinical response.

Daillere et al. announced two bacterial species, *E. hirae* and *Barnesiella intestinihominis*, that play an important role in determining the antitumoral efficacy of cyclophosphamide (CTX), an anti-cancer immunomodulatory agent (Daillère et al. 2016). *E. hirae*, a gram-positive bacteria residing in the small intestine, was found to selectively restore the CTX-mediated antitumor effects in ATB-treated mice and improved the cognate anticancer immune response. *E. hirae* specifically induced the systemic effector pathogenic Th17 (pTh17) cell responses related to MHC class I-restricted cytotoxic T cells (CTL) and significantly decreased the level of Foxp3^+^CD25^+^CD4^+^TILs resulting in increased intratumoral CTL/T regulatory (Treg) cell ratio. The gram-negative *B. intestinihominis*, residing in the colon, enhanced the systemic polyfunctional CD4^+^ Tc1 and CD8^+^ Th1 cell responses by augmenting the proportions of intratumoral IFN-ɣ-producing ɣdTILs while decreasing IL-17-producing ɣdTILs. In the spleen, *E. hirae* and *B. intestinihominis*, representing oncomicrobiotics (OMBs), facilitated the accumulation of effector CD8^+^ T cells by inverting the CD4/CD8 T cell ratio. Repetitive gavages of either OMB ameliorated the CTX activity against MCA-205 fibro-sarcoma. Gavages with *B. intestinihominis* were also efficacious against Ret melanoma. Daillere and colleagues found that IFN-ɣ neutralization with unaltered IL-17 or CD8^+^ T cell depletion critically debilitated the anticancer efficacy of the two commensals against MCA-205. Therefore, these OMBs exhibit the effect of an adjuvant on the tumoral immune responses, enhancing the efficacy of CTX. The authors also discovered that the immune-dependent anti-sarcoma effects of CTX were considerably enhanced in nucleotide oligomerization domain 2-deleted (*Nod2*^*−/−*^) mice. CTX-treated sarcoma growing in *Nod2*^*−/−*^ mice showed lower number of Treg cells and higher number of IFN-ɣ-producing ɣdTILs compared to sarcoma growing in a wild type environment. Hence, NOD2 receptors were suggested to function as “gut immune checkpoints” that limited the immunogenicity of *E. hirae* and *B. intestinihominis*. Therefore, NOD2 hinders the accumulation of the two OMBS and restricts their proapoptotic effects on the epithelial cells. The study also concluded the immune response of memory Th1 toward *E. hirae* or *B. intestinihominis* to govern the progression-free survival in cancer patients.

Gopalakrishnan et al. investigated the oral and gut microbiome of melanoma patients who received anti-PD-1 immunotherapy based on a previous research finding that reported gut microbiome to modulate the tumor response to ICI treatment (Gopalakrishnan et al. 2018). They analyzed the patient’s fecal microbiome samples and assessed the relative abundance of bacteria in the R and NR groups. The bacterial population was significantly different between the two groups. The R group exhibited substantially higher alpha diversity and higher frequency of bacteria belonging to the *Ruminococcaceae* family (*p* < 0.01) than the NR group. This favorable gut microbiome exhibited improved antitumor immunity in R patients and fecal transplanted germ-free mice. High abundance of *Faecalibacterium* sp. in the R group heightened the antigen presentation and T cell function in the tumor microenvironment, thereby, enhancing the antitumor immune responses. On the other hand, high relative abundance of *Bacteroidales* was associated with decreased antitumor immune responses owing to the diminished antigen presentation capacity. While increased activation of CD8^+^ T cells was observed in R-FMT mice, the spleen of NR-FMT mice exhibited increased levels of regulatory CD4^+^FoxP3^+^ T cells and CD4 IL-17 T cells. This suggests that FMT from NR resulted in impaired host immune responses. The study emphasized on the therapeutic potential of regulating microbial composition in patients undergoing ICI therapy. While Gopalakrishnan et al., Routy et al., and Matson et al. studied the link between gut microbiome and efficacy of anti-PD-1 mAb in solid cancers (Gopalakrishnan et al. 2018; Matson et al. 2018; Routy et al. 2018), Matson et al.’s study was an exception owing to the different method used to identify the R and NR patients (Matson et al. 2018).

In a similar study, Tanoue et al. studied 11 bacterial strains that were positively associated with the frequency of IFN-ɣ-producing CD8 T cells. These bacterial strains, isolated from healthy human donor feces, were mixed inoculated into germ-free mice (GF+11-mix mice) (Tanoue et al. 2019). A substantial increase in IFN-ɣ^+^ CD8 T cells was found in diverse genetic backgrounds, with relative enrichment for the T cell receptor (TCR) Vβ6^+^ and Vβ8^+^ subsets. GF+11-mix mice also exhibited induction of CD4 T cells, increasing the frequencies of colonic Th17 and Th1 cells as well. Among these 11 strains, 7 were *Bacteroidales*, which when mixed and inoculated into germ-free mice (7-mix) were incapable of inducing IFN-ɣ + CD8 T cells. The mix of the other 4 strains, which were non-*Bacteroidales*, although exhibited a better induction capacity, but still was not sufficient to yield the complete inductive effect of the 11-mix. Hence, it was suggested that the 11 strains, including the 7 *Bacteroidales* as supporting strains and the 4 non-*Bacteroidales* as effector elements, formed a consortium. The upregulation of chemokines CXCl9, CXCl10, and IFN-inducible genes in the colonic epithelial cells by the colonization of the 11-mix suggested that the recruitment and accumulation of IFN-ɣ + CD8 T cells can be promoted by active colonization near epithelial cells, establishing an IFN-ɣ-mediated feed-forward loop. Subcutaneous engraftment of GF+11-mix mice with MC38 adenocarcinoma cells showed significantly improved anti-PD-1 treatment efficacy, compared to that in germ-free mice and in mice with strains that do not induce IFN-γ^+^ CD8 T cells. This therapeutic effect was a result of increased IFN-γ^+^ CD8 TILs, as the depletion of CD8 T cell by antibody notably reduced the efficacy. Interestingly, the colonization of 11-mix even suppressed the tumor growth without anti-PD-1 treatment. Altogether, this data demonstrates that the 11 strains with non-inflammatory immunomodulatory activity enhance both ICI-mediated and spontaneous anti-tumor immunity in a CD8 T cell-dependent manner. Evaluating the abundance of the 11 strains using metagenomic datasets, it was observed that majority of the strains were rare and low-abundance components of the human microbiota. The low-abundance trend was also established in both R and NR with melanoma receiving ICI treatment in the studies by Gopalakrishnan et al. (2018), Routy et al. (2018) and Matson et al. (2018).

Likewise, Pushalkar et al. established a specific gut microbiome that is linked to the progressive pancreatic oncogenesis in mice (Pushalkar et al. 2018). Analyzing the gut microbiome using 16S rRNA fluorescence in-situ hybridization (FISH) and qPCR, it was observed that both the mouse and human pancreatic ductal adenocarcinoma (PDA) exhibit significantly greater bacterial abundance compared to the normal pancreas. The most abundant of the 13 phyla that were detected in the human PDA were *Proteobacteria*, *Bacteroidetes*, and *Firmicutes*. The PDA-associated microbiome differentially activated selective Toll-like receptors (TLR) in order to create a tolerogenic immune program. The microbiome was observed to render suppressive effects on the T-cell activation when polarized on macrophages. However, in the absence of TLR signaling, this effect was attenuated. *Bifidobacterium pseudolongum*, differentially abundant in both gut and tumor, was also reported to accelerate oncogenesis in a TLR-dependent manner. Further, the macrophages polarized by cell-free extracts of *B. pseudolongum* upregulated the tolerogenic cytokine IL-10. Ablation of the gut bacteria by oral administration of antibiotics resulted in an increased activation of Th1 differentiation of both of CD4^+^ and CD8^+^ T cells and decreased immune-suppressive cells. Thus, the ablation was associated with decrease in immune-suppressive CD206^+^ M2-like tumor-associated macrophages (TAM), an adjuvant to increase M1-like TAMs, which express MHC II, CD86, tumor necrosis factor alpha (TNF- α), IL-12, and IL-6. High expression of T-BET, TNF-α, and IFN-ɣ indicated an enhanced cytotoxic phenotype of CD8^+^ T cells**.** The authors, thus, suggested bacterial ablation to create a synergistic efficacy with PD-1-directed therapies. Bacterial transfer from PDA-bearing hosts reversed the protective effect of microbial ablation against PDA. However, the reversion was not evident in a transfer from control-host; thus, emphasizing on the pro-tumorigenic effect of PDA-associated microbiome.

### Metabolites as a mechanism of modulating host cytokines and anticancer therapeutics

While the specific functions and molecular mechanism of the gut microbiome are yet largely unidentified, Lee et al. explored a few of the strain-specific mechanisms of how the microbiome affects cancer development and the efficacy of anti-cancer therapy (Lee et al. 2021). Lee et al. found that the increase in anticancer immunity due to *B*. *bifidum* strain-specific enhancement of anticancer immunity is a result of IFN-γ signaling. In addition, the *B*. *bifidum*-induced IFN-γ production was enhanced by the increase in serum metabolites and peptidoglycan biosynthesis.

Lee et al. collected intestinal tissues from tumor mice treated with anti-PD-1 plus synergistic *B*. *bifidum* strains (B. bif_K57) or non-synergistic strains (B. bif_B06). By performing RNA sequencing intestinal tissues, they were able to observe a notably higher number of genes associated with Gene Ontology terms “lymphocyte activation”, “interferon-gamma production” and “positive regulation of interferon-gamma production” in mice treated with anti-PD-1 with B. bif_K57 compared to those treated with B. bif_B06. Serum metabolic profiling showed that the concentrations of l-tryptophan, uric acid, and *N*-acetyl zonisamide were higher in mice treated with anti-PD-1 plus B. bif_K57 compared to mice treated with anti-PD-1 plus B. bif_B06. It was also found that in vitro, the addition of l-tryptophan increased IFN-γ production from activated CD8+ T cells. Mice treated with anti-PD-1 plus B. bif_B57 exhibited the lowest serum lipid levels, followed by mice treated with anti-PD-1 plus B. bif_B06 and then anti-PD-1. This, in line with a previous study that demonstrated that lipid challenge leads to the decrease in T cell secretion of IFN- γ (Guerrero-Ros et al. 2020), shows that lowering the lipid level is an example of a mechanism by which the microbiome modulates cytokines.

Lee et al. also discovered that the peptidoglycan levels were significantly higher in B. bif_K57 compared with B. bif_B06. MurE and GlmM, which are the genes associated with the peptidoglycan biosynthetic process, were expressed more strongly in anti-PD-1 plus B. bif_K57-treated mice stools compared to anti-PD-1 plus B. bif_B06-treated mice. In patients with NSCLC, the stool samples of patients containing the *B*. *bifidum* exhibited enriched peptidoglycan biosynthesis pathway, compared to bifidum-free patients, demonstrating the effect of peptidoglycans on immune modulation.

## Conclusion

The findings of the different studies discussed in this review highlighted a strong correlation between the gut microbiota and immune system. The significant role of the gut microbiome in regulating the antitumor activity of ICIs was also elucidated. Notably, species belonging to the genera *Akkermansia* (Routy et al. 2018; Xu et al. 2020)*, Enterococcus* (Daillère et al. 2016; Matson et al. 2018; Routy et al. 2018), *Bifidobacterium* (Matson et al. 2018; Pushalkar et al. 2018; Sivan et al. 2015) and *Ruminococcaceae* (Gopalakrishnan et al. 2018; Tanoue et al. 2019) were found to improve the immune responses and increase the efficacy of PD-1 blockade therapy. Interestingly, the genus *Bacteroides* has been found to induce biphasic effects. While some strains of *Bacteroides* increase the response to CTLA-4 blockade (Vétizou et al. 2015), other strains of this genus are associated with negative therapeutic efficacy of PD-1 antibody immunotherapy (Xu et al. 2020). Gut microbial changes induce changes in the expression of cytokines and immune cells, resulting in differential therapeutic responses. Particular cytokines, IFN-ɣ (Daillère et al. 2016; Garris et al. 2018; Matson et al. 2018; Routy et al. 2018; Tanoue et al. 2019; Xu et al. 2020), IL-12 (Garris et al. 2018; Routy et al. 2018; Vétizou et al. 2015), and IL-17 (Daillère et al. 2016; Gopalakrishnan et al. 2018), have been reported as the key cytokines that are used by certain bacterial strains to affect the immunomodulation. Additionally, immune cells such as the CD8^+^ T cells (Matson et al. 2018; Pushalkar et al. 2018; Sivan et al. 2015; Tanoue et al. 2019) and CD4^+^ T cells (Routy et al. 2018; Gopalakrishnan et al. 2018; Daillère et al. 2016; Pushalkar et al. 2018) have also been identified to be responsible for the responsiveness to ICP blockade therapy. The mechanism behind the strain-specific enhancement of anticancer immunomodulation was studied by Lee et al., in which they found that the IFN-γ signaling was the key to the B. *bifidum* strain-specific anticancer immunity (Lee et al. 2021). More importantly, they discovered that the increase in serum l-tryptophan, along with other serum metabolites, and peptidoglycan biosynthesis augmented the *B*. *bifidum*-induced IFN-γ production.

While some cytokines reveal the possible mechanism by which specific bacterial strains confer increased efficacy of ICI therapy, future studies are warranted to elucidate the detailed molecular mechanism of the mutual interaction. In addition, a more comprehensive analysis of the microbial composition associated with anticancer response will be necessary to better understand the link between the microbiome and their immunomodulatory effects. The isolated strains carry significant biotherapeutic potentials: they can be utilized as biomarkers to predict the efficacy of ICI and can also be applied to treat cancer and other related diseases.

Bacterial components are well-known pathogen-associated molecular patterns (PAMPs) that induce the innate immune system via the TLRs. However, antitumor immunity is usually exerted by the adaptive immune system, such as CD8 cytotoxic T cells specific for cancer peptides. Thus, it is important to clarify how the PAMPs activate cancer-specific adaptive immunity. Furthermore, while certain bacterial species enhance the efficacy of ICIs, some repress its efficacy, implying that the antitumor activity of the bacteria may not stem from the simple activation of the innate immune system by common PAMPs, such as lipopolysaccharide (LPS). More detailed understanding on how gut microbiota controls the systemic immune system is essential for better understanding and development of efficient therapeutics against cancers that lack efficient treatment strategies.
